# Intestinal Inflammation in Children with Cystic Fibrosis Is Associated with Crohn’s-Like Microbiota Disturbances

**DOI:** 10.3390/jcm8050645

**Published:** 2019-05-10

**Authors:** Raphaël Enaud, Katarzyna B. Hooks, Aurélien Barre, Thomas Barnetche, Christophe Hubert, Marie Massot, Thomas Bazin, Haude Clouzeau, Stéphanie Bui, Michael Fayon, Patrick Berger, Philippe Lehours, Cécile Bébéar, Macha Nikolski, Thierry Lamireau, Laurence Delhaes, Thierry Schaeverbeke

**Affiliations:** 1 Centre de Recherche Cardio-Thoracique de Bordeaux, INSERM, University Bordeaux, U1045, F-33000 Bordeaux, France; michael.fayon@chu-bordeaux.fr (M.F.); patrick.berger@u-bordeaux.fr (P.B.); laurence.delhaes@chu-bordeaux.fr (L.D.); 2 CRCM Pédiatrique, CHU Bordeaux, CIC 1401, F-33000 Bordeaux, France; haude.clouzeau@chu-bordeaux.fr (H.C.); stephanie.bui@chu-bordeaux.fr (S.B.); thierry.lamireau@chu-bordeaux.fr (T.L.); 3 Fédération Hospitalo-Universitaire FHU, ACRONIM, F-33000 Bordeaux, France; thomas.barnetche@chu-bordeaux.fr (T.B.); cecile.bebear@u-bordeaux.fr (C.B.); thierry.schaeverbeke@chu-bordeaux.fr (T.S.); 4 Bordeaux Bioinformatics Center, University Bordeaux, F-33000 Bordeaux, France; aurelien.barre@u-bordeaux.fr (A.B.); macha@labri.fr (M.N.); 5 Laboratoire Bordelais de Recherche en Informatique, CNRS, University Bordeaux, UMR 5800, F-33400 Talence, France; 6 Service de Rhumatologie, CHU Bordeaux, F-33000 Bordeaux, France; 7 INSERM, MRGM, University Bordeaux, U1211, F-33000 Bordeaux, France; christophe.hubert@u-bordeaux.fr; 8 PGTB, University Bordeaux, F-33000 Bordeaux, France; 9 BIOGECO, INRA, University Bordeaux, F-33610 Cestas, France; marie.massot@inra.fr; 10 INRA—Bordeaux Aquitaine Centre, University Bordeaux, USC EA 3671, Infections Humaines à Mycoplasmes et à Chlamydiae, CHU Bordeaux, F-33000 Bordeaux, France; thomasbazin@club-internet.fr; 11 BaRITOn, INSERM, University Bordeaux, UMR1053, CHU Bordeaux, F-33000 Bordeaux, France; philippe.lehours@chu-bordeaux.fr

**Keywords:** cystic fibrosis, gut microbiota, intestinal inflammation, fecal calprotectin, dysbiosis index

## Abstract

Cystic fibrosis (CF) is a systemic genetic disease that leads to pulmonary and digestive disorders. In the majority of CF patients, the intestine is the site of chronic inflammation and microbiota disturbances. The link between gut inflammation and microbiota dysbiosis is still poorly understood. The main objective of this study was to assess gut microbiota composition in CF children depending on their intestinal inflammation. We collected fecal samples from 20 children with CF. Fecal calprotectin levels were measured and fecal microbiota was analyzed by 16S rRNA sequencing. We observed intestinal inflammation was associated with microbiota disturbances characterized mainly by increased abundances of *Staphylococcus*, *Streptococcus,* and *Veillonella dispar*, along with decreased abundances of *Bacteroides*, *Bifidobacterium* *adolescentis*, and *Faecalibacterium prausnitzii*. Those changes exhibited similarities with that of Crohn’s disease (CD), as evidenced by the elevated CD Microbial-Dysbiosis index that we applied for the first time in CF. Furthermore, the significant over-representation of *Streptococcus* in children with intestinal inflammation appears to be specific to CF and raises the issue of gut–lung axis involvement. Taken together, our results provide new arguments to link gut microbiota and intestinal inflammation in CF and suggest the key role of the gut–lung axis in the CF evolution.

## 1. Introduction

Cystic fibrosis (CF) is a genetic disorder caused by mutations in the Cystic Fibrosis Transmembrane Conductance Regulator gene (CFTR), leading to viscous secretions accumulating on epithelial surfaces in both the lungs and the gastrointestinal tract [1]. In recent decades, improved patient care in the management of pulmonary disease has led to an older CF population with new complications, including intestinal disorders [2]. However, some CF gastrointestinal complications such as chronic inflammation, gut microbiota disruption, and increased risk of gastrointestinal malignancies remain poorly understood [3,4,5,6].

Gut microbiota has been recently shown to be associated with the human health and diseases including CF [7]. Its composition is clearly different in CF patients, with a decrease in specific bacteria such as *Bifidobacterium* spp., *Eubacterium* spp., *Clostridium* spp., and *Faecalibacterium prausnitzii*, and the emergence of opportunistic pro-inflammatory bacteria such as *Escherichia coli* and *Eubacterium biforme* [4,8,9,10,11]. In CF, this disturbed microbiota, usually named “dysbiosis”, stems from multiple factors including hydro electrolytic disruptions of the intestinal secretions, slower gastrointestinal transit time [12], drug uses, impaired innate immunity in the gut [13,14], and hypercaloric diet [15,16], and appears to be correlated with the severity of the CFTR mutations [11].

Furthermore, chronic intestinal inflammation is present in the majority of CF patients, even in the absence of digestive symptoms [17]. Inflammation is characterized by an infiltrate of the lamina propria by mononuclear cells expressing inflammation markers (such as ICAM-1, CD-25, IL-2, IFNγ) associated with Crohn’s-like endoscopic lesions of the mucosa (edema, erythema, or ulcerations) [3,18,19]. To better assess intestinal inflammation, Bruzzese et al. have adapted in CF the fecal calprotectin measurement, previously used as a non-invasive digestive inflammation biomarker in inflammatory bowel disease (IBD) [20]. Fecal calprotectin level is significantly higher in CF patients compared to healthy subjects [20,21,22].

The pathophysiology of intestinal inflammation is still unclear in CF. Very few studies have focused on the link between intestinal microbiota composition and inflammation in CF, while this link is well documented in IBD [23]. To our knowledge, only one study using next-generation sequencing (NGS) of microbiota was dedicated to this issue and showed a positive correlation between *E. coli* abundance and intestinal inflammation in CF children [24]. A recent study focused on the evolution of intestinal microbiota and inflammation in CF patients treated with ivacaftor, a CFTR-modifying therapy. An absence of intestinal inflammation was significantly associated with an increased *Akkermansia* abundance [25].

As previous studies focused on mucosal inflammation and microbiota in the gastrointestinal tract of CF patients were highly limited [22,24], our aim was to investigate links between gut microbiome and intestinal inflammation using NGS approach plus fecal calprotectin measurements, in a pediatric CF population.

## 2. Materials and Methods

### 2.1. Study Design, Sample Collection, and Ethics Statement

Our observational prospective study took place at the Children’s Hospital of Bordeaux from November 2015 to May 2018. The inclusion criteria were patient over 3 years of age with well-documented CF associated with exocrine pancreatic insufficiency. The exclusion criteria were an ongoing enrollment in therapeutic protocols, antibiotics, or probiotics courses during the two months prior the inclusion or patients after organ transplantation.

At the inclusion visit patients’ stool samples were collected and stored at −80 °C until use. In parallel, patient clinical status was documented using demographic data, nutritional status assessed by the Body Mass Index expressed as percent of the standard normalized by age (%BMI), respiratory capacity measured by Forced Expiratory Volume in 1s expressed as percent predicted (%FEV_1_), and microbial pulmonary colonization along with previous intravenous (IV), oral, or inhaled antibiotic courses. 

In addition, questionnaires focused on standardized assessments of quality of life (PedsQL^TM^ 4.0 Generic Core Scale) and of digestive symptoms (PedsQL^TM^—Gastrointestinal Symptoms Scales 3.0) were provided at no charge via the ePROVIDE™ online distribution process and filled for each child. These questionnaires, validated for different age groups, include a self-assessment and a parental evaluation. A score negatively correlated with the presence of symptoms was assigned from 0 to 100 to each item based on the collected responses. An average score was calculated for the main questionnaire sections. 

Finally, long-term evolution based on clinical monitoring two years after inclusion was recorded for each child, based on %BMI and %FEV_1_ values at the follow-up visit, plus the corresponding variations (estimated by the difference between %BMI or %FEV_1_ measures at two years and inclusion), and the number of antibiotic courses during this period (IV, oral, inhaled, or any mode of administration combined) (Table 1 and Supplementary Table S1).

The present study was approved by the regional ethical committee “CPP Sud-Ouest et Outremer III” (DC 2015/129). Informed consent was sought from study participants and their parents.

### 2.2. Measurements of Fecal Calprotectin

The fecal calprotectin assay was carried out using the GHSA kit (Eurobio, Courtabœuf, France). In the absence of a specific threshold in CF, we applied a cut-off of 250 μg/g, recently validated to predict an inflammatory flare associated with endoscopic lesions in IBD [26,27]. 

### 2.3. Microbiota Analysis

DNA extraction was performed using DNeasy Blood & Tissue kit (Qiagen, Hilden, Germany) according to the manufacturer’s protocol. The DNA samples were the used for V4 region of the 16S rRNA gene sequencing as previously described [28]. For specific analysis of *S. oralis* and total bacteria population, droplet digital PCR (ddPCR) on the fecal DNA was performed, as described in the Supplemental Information.

### 2.4. Sequencing and Bioinformatics Analysis

Next-generation sequencing was performed using Illumina MiSeq sequencer, and bioinformatic analysis as described previously [28]. Alpha and beta diversity indexes were assessed using raw Operational Taxonomic Unit (OTU) occurrence counts and a Non-Metric Multidimensional Scaling (NMDS) ordination method with Bray-Curtis distance metric implemented by R package phyloseq, respectively. Subsequent OTU filtering and analyses were performed with MicrobiomeAnalyst (http://www.microbiomeanalyst.ca) [29]. OTUs were normalized by total sum scaling. Two groups of patients were defined with low (<250 µg/g) and high (>250 µg/g) fecal calprotectin level and used for DESeq2 [30] (Supplementary Table S2) and LEfSe [31] (Supplementary Table S3) analyses to find differentially present taxa and microbiota markers, respectively. Raw data have been deposited in the European Nucleotide Archive (ENA) sequence read archive (ENA accession number PRJEB28609).

### 2.5. Microbial Dysbiosis Index Evaluation

We estimated the Microbial Dysbiosis index (MD-index; Supplementary Table S4), a ratio between relative abundance of increased and decreased bacteria recently proposed in Crohn’s disease (CD) [32]. 

### 2.6. Statistical Analysis

Differentially present taxonomic nodes between groups of patients were calculated using DESeq2 approach and a False Discovery Rate (FDR) < 0.05. LEfSe method was used to identify metagenomic biomarkers [31]. Nonparametric Wilcoxon–Mann–Whitney test was used to compare quantitative variables between groups. Correlations were calculated using Spearman method. Statistical analysis was performed with R studio program (version 1.1.453 for Windows^TM^); a *p*-value < 0.05 was considered indicative of statistical significance.

## 3. Results

**Intestinal inflammation is associated with more previous intravenous antibiotic courses in CF.** Twenty children with CF between 6 and 14 years of age were included; their main demographic characteristics, clinical and microbiological data are summarized in Table 1. Among them, we identified seven (35%) children (mean of age at 8.0 ± 3.0 years old, Table 1) with significant intestinal inflammation (fecal calprotectin > 250 µg/g, with levels ranged from 300 to 1800 µg/g). Age of patients, mutation severities, %BMI and %FEV_1_ were not significantly different between children without and with intestinal inflammation (Table 1). In addition, no complaint recorded in questionnaires could discriminate inflammatory status of children (Table 1). However, children with intestinal inflammation have received significantly more intravenous antibiotic courses before inclusion (*p* = 0.04 adjusted with age, Table 1). 

**Figure 1:**
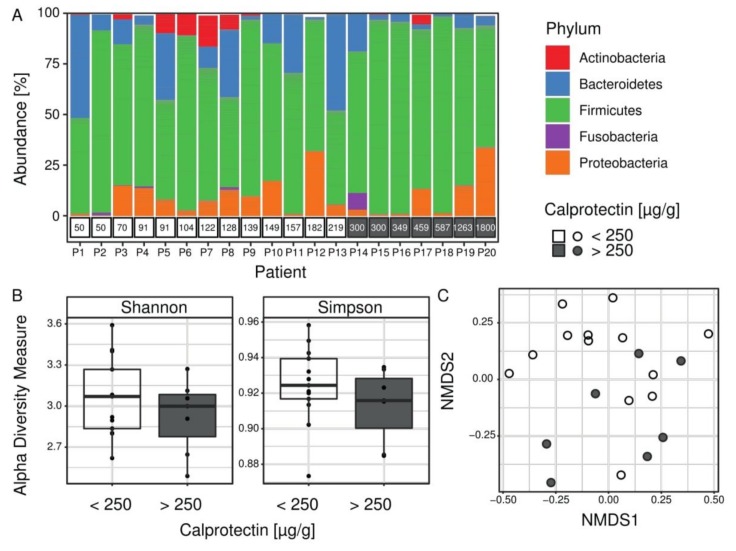
**Microbiota composition in the Cystic Fibrosis (CF) cohort**. (**A**) Proportions of bacteria from the five most abundant phyla colored according to the legend. Calprotectin measurements per patient are shown in boxes below the bar plot. The proportion of Firmicutes was significantly higher in microbiota profiles of children with intestinal inflammation (gray boxes). (**B**) Alpha diversity values for all patients (*n* = 20) are shown as points and summarized as boxplots for each group. Both Shannon and Simpson alpha indices measure microbial diversity within sample, and they were not significantly different between children with and without intestinal inflammation (Wilcoxon-–Mann–Whitney test). (**C**) Beta diversity (NMDS), which assesses differences in microbial composition between samples using a NMDS ordination method with Bray–Curtis distance metric, showed a partial separation of samples of patients with intestinal inflammation.

**CF children with intestinal inflammation are distinguished by their intestinal microbiota composition.** The microbiota composition from 20 fecal samples was estimated using targeted 16S rRNA gene sequencing. After bioinformatic analysis, the median number of high-quality reads per patient was 421,720 (from 69,534 to 2,500,796). Taxonomic assignment was used to compare the profiles of patients’ microbiota (Figure 1). At the phylum level, the proportion of Firmicutes was significantly higher in microbiota profiles of children with intestinal inflammation (on average 81% vs. 65% respectively for children with and without intestinal inflammation, FDR = 0.0078) (Figure 1A). To evaluate species diversity in patients’ microbiome we calculated alpha diversity indices. The Shannon and Simpson indices are two complementary approaches to alpha diversity, sensitive to changes in abundance of the rarest or the most abundant OTUs, respectively. Alpha diversity indexes were not significantly different between children with and without intestinal inflammation (Figure 1B). However, the beta diversity analysis (NMDS) reflecting the variation of microbiome between samples, showed partial separation of the patients with intestinal inflammation (Figure 1C). 

**Figure 2:**
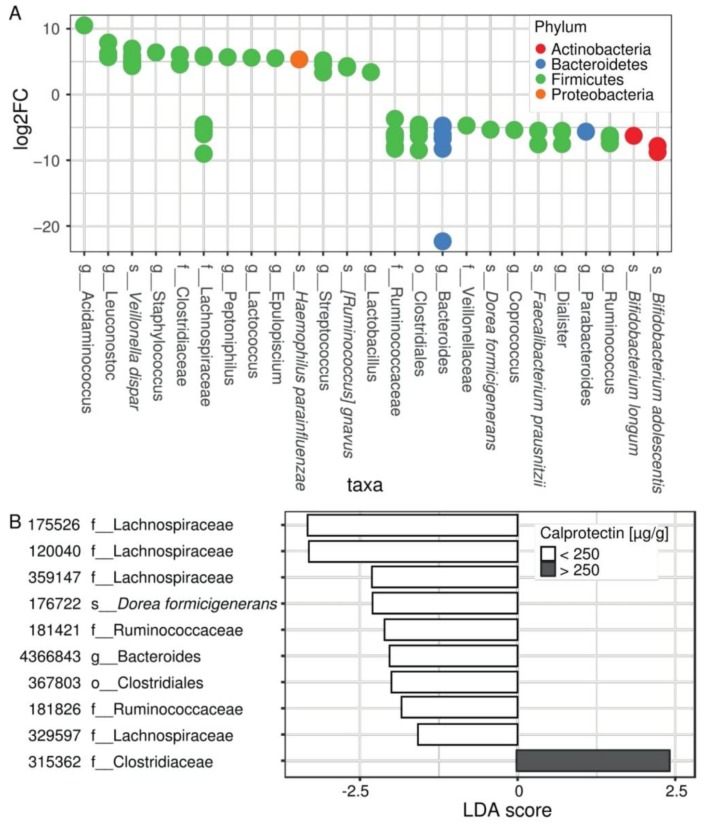
**The composition of the microbiota differs according to the inflammation status of CF patients.** (**A**) Differential abundance analysis (DESeq) assessing OTU significantly changed in the microbiome of patients with intestinal inflammation, compared with patients without intestinal inflammation. Each circle represents one of 80 significant OTUs colored by a phylum according to the legend. OTUs are collapsed to 25 taxa represented on x axis and ordered by decreasing log of fold change. For full results see Supplementary Table S2. (**B**) LEfSe analysis showing OTUs distinguishing patients without and with intestinal inflammation (*p*-value < 0.01) and confirming DESeq2 results. For full results see Supplementary Table S3.

Differential expression (DESeq2) analysis revealed 80 distinctive OTUs that belonged to 25 unique taxonomic nodes differentially present between patients according to their intestinal inflammation status (Figure 2A, Supplementary Table S2). Among them, increased abundances of *Acidaminococcus* spp., *Staphylococcus* spp., *Streptococcus* spp., and *Veillonella dispar,* along with decreased abundances of *Bacteroides* spp., *Ruminococcus* spp., *Coprococcus* spp., *Dialister* spp., *Parabacteroides* spp., *Bifidobacterium* spp., *Dorea formicigenerans,* and *Faecalibacterium prausnitzii* were observed in children with fecal calprotectin higher than 250 µg/g (Figure 2A). 

Using LEfSe analysis (Supplementary Table S3), we identified numerous taxonomic nodes that can predict intestinal inflammation in our young CF population. It confirmed the results of DESeq2 at genus levels regarding *Staphylococcus, Streptococcus*, *Bacteroides*, *Ruminococcus, Coprococcus, Dialister*, and *Parabacteroides,* and at the species level regarding *Veillonella dispar*, *Bifidobacterium adolescentis, Dorea formicigenerans*, and *Faecalibacterium prausnitzii.* In addition, some OTUs belonging to the families Lachnospiraceae and Ruminococcaceae appear to be differentially correlated to the inflammatory patient status (Figure 2B, results with *p*-value < 0.01). 

**Figure 3:**
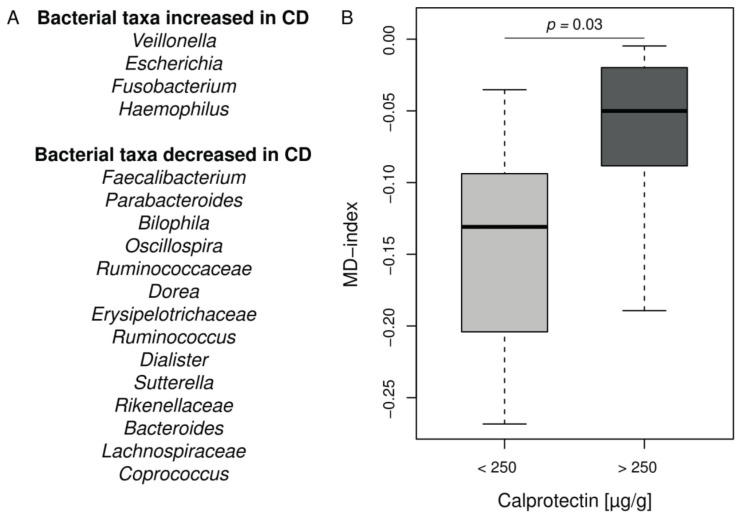
**MD-index distribution in CF cohort according to the intestinal inflammation status.** (**A**) Bacterial taxa contributing to the MD-index according to [32]. (**B**) Boxplot of MD-index values for patients separated into groups according to calprotectin level. Patients with CF intestinal inflammation have a significant higher MD-index (Wilcoxon–Mann–Whitney test, *p* = 0.03). For full results see Supplementary Table S4.

**CF intestinal inflammation and microbiota exhibit similarities with IBD.** In order to analyze deeper the CF intestinal microbiota changes in our cohort, we applied the MD-index, recently designed and validated in CD [32]. This index is based on a ratio between given bacterial taxa known to be increased or decreased in CD listed in Figure 3A. The MD-index was significantly higher in the CF children with intestinal inflammation compared to the group without inflammation (*p* = 0.03) (Figure 3B and Supplementary Table S4). It was not correlated to age, %BMI, or %FEV_1_ in our cohort. 

**Figure 4:**
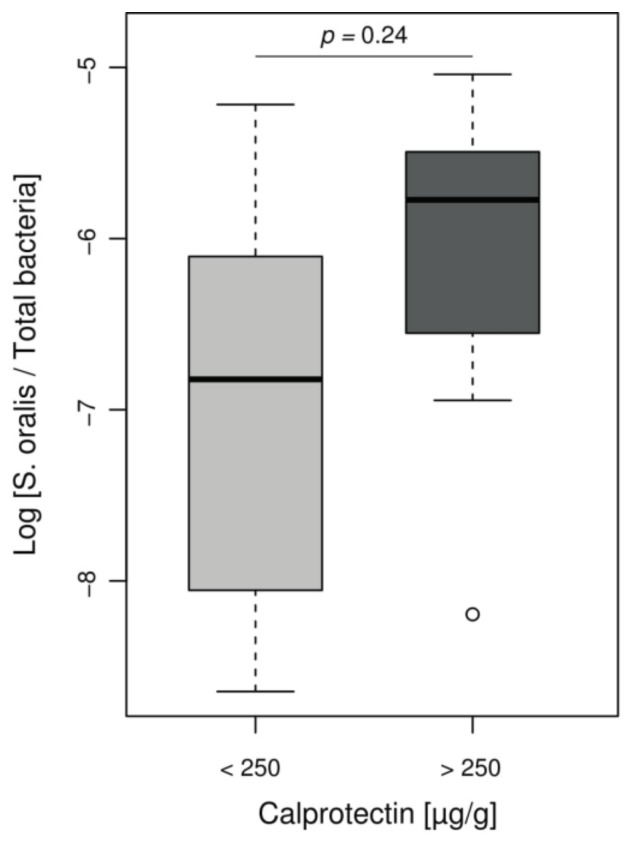
**Relative abundance of *S. oralis* assessed by ddPCR according to the inflammation status of CF patients.** Boxplot of values representing relative proportion of *S. oralis* in patients’ microbiomes, separated into groups according to calprotectin level. Among *Streptococcus* found in CF children, there was a notable proportion of *S. oralis* but without significant difference between children with or without intestinal inflammation (Wilcoxon–Mann–Whitney test, *p* = 0.24).

However, *Streptococcus* genus, increased in our inflammatory patient group, is not usually associated with IBD and is not included in the MD-index. Given the role of *Streptococcus mitis* group in CF lung disease evolution [33,34] and difficulties in distinguishing this group from other *Streptococci* by NGS, we assessed the presence of mitis group species by using ddPCR that specifically targeted *Streptococcus oralis*. We observed a non-significant increase log ratios of *S. oralis* per total bacteria in children with intestinal inflammation, compared to children with low calprotectin levels (ratio median −5.8 and −6.8, respectively, *p* = 0.24) (Figure 4). 

**Intestinal inflammation at the inclusion is associated with increased antibiotic use over the next two years.** We compared the clinical evolution at two years between children without and with intestinal inflammation. While intestinal inflammation was not predictive of evolution of %FEV_1_ or %BMI at two years (Table 1), children with intestinal inflammation at the inclusion had more antibiotic treatments over the next two years, all routes of administration combined (*p* = 0.02). Surprisingly, this discrepancy appears to be more related to inhaled antibiotic courses (*p* = 0.02) than to intravenous (*p* = 0.06) or oral (*p* > 0.05) antibiotic therapies (Table 1).

## 4. Discussion

Intestinal microbiota disturbances and intestinal inflammation are now widely accepted as an integral part of CF. With the increased life expectancy of patients, the management of these digestive disorders is becoming a topical subject, even if the corresponding physiological mechanisms are not yet well understood. In fact, links between intestinal microbiota and inflammation in CF have been suggested directly or indirectly by a limited number of studies [5]. In one of the few CF studies analyzing gut microbiota by NGS according to inflammation, intestinal inflammation was associated with an increase of *E. coli* abundance [24]. In another study, gastrointestinal mucosal lesions recorded by capsule endoscopy was positively correlated with Firmicutes and negatively correlated with Bacteroidetes [3]. Indirectly, probiotic intake or antibiotic courses in CF patients were able to decrease intestinal inflammation [20,35]. 

In order to refine the relationships between intestinal microbiota composition and inflammation in the CF, we compared intestinal microbiota profiles from young CF patients, according to their intestinal inflammatory status evaluated by fecal calprotectin. Despite the limited number of children included (*n* = 20) and their young age (mean of age at 8.0 ± 3.0 y.o. in Table 1), we identified a notable proportion of children (35%) with significant intestinal inflammation and microbiota disturbances (Figure 1 and Figure 2). In this group, fecal calprotectin levels ranged from 300 to 1800 µg/g, rates comparable to those seen in IBD [26]. We observed increased abundances of *Staphylococcus* spp., *Streptococcus* spp., and *Veillonella dispar,* along with decreased abundances of *Bacteroides* spp., *Ruminococcus* spp., *Coprococcus* spp., *Dialister* spp., *Parabacteroides* spp., *Bifidobacterium adolescentis,* and *Faecalibacterium prausnitzii* in children with fecal calprotectin higher than 250 µg/g (Figure 3). We did not find significant increased *E. coli* abundances, as previously reported in a cohort of younger CF children (median age at 10 months) [24]. Our results were confirmed using LEfSe (Supplementary Table S3). On the whole, these microbiota disturbances exhibited numerous similarities with the well described IBD microbiota [23,32,36]. Interestingly, the MD-index, which is positively correlated with the clinical disease activity of CD [32], was significantly higher in our group of CF children with high fecal calprotectin levels. 

Decrease of *B. adolescentis* and *F. prausnitzii* in group of children with intestinal inflammation confirm the results recently published in CF [22] and are congruent with data exploring in vitro anti-inflammatory proprieties of bacteria. *Bifidobacterium adolescentis* inhibits inflammatory responses in intestinal epithelial cells, by limiting the lipopolysaccharide-induced inflammatory response and the TNF-α production [37]. Furthermore, the intestinal inflammation in our cohort was associated with an under-representation of *F. prausnitzii* (Figure 2B), a strain well investigated in IBD for its anti-inflammatory properties and considered as a viable marker of health [36]. 

Taken together, these results raise the question of the causal relationship between microbiota composition and inflammation: does a decrease of anti-inflammatory bacteria lead to inflammation or does it result from this inflammation as a basic consequence of anti-inflammatory bacteria decrease? The answer to this question is still a matter of debate in CF as well as IBD [38]. Keeping in mind that the causal relation is probably bidirectional, few published data suggest the existence of a pro-inflammatory microbiota. Experiments using a murine model of colitis showed that disease can be transmitted from a genetically modified mouse to a wild mouse by a microbiota transfer [39]. In CF, oral antibiotic or probiotic exposures were shown to reduce intestinal inflammation [20,35]. A higher MD-index in our inflammatory subpopulation lets us suggest that these patients may have a more pronounced “pro-inflammatory” microbiota (Figure 2A and Figure 3B). This subpopulation exhibited also significantly more Firmicutes and had previously received more IV antibiotics, which is a known factor leading to an increase of Firmicutes (Table 1 and Figure 1A) [8]. Moreover, the young age of our cohort together with the microbiota disturbances observed in CF from the first weeks of life [9,40] seem to indicate that CF gut microbiota changes is inherent to the disease rather than a basic consequence of inflammation and/or antibiotic use. 

More recently, the human microbiome (which includes the gut microbiome but also the other body sites) has emerged as a complex interconnected entity leading to the popularization of the concepts of gut–brain and gut–lung axes [41,42]. The gut–lung axis is of major interest in CF, as patients colonize their digestive tract with microorganisms from oral or respiratory tracts by sputum swallowing [43]. Bacteria found in gastric fluid were correlated with CF sputum microbiota composition, especially regarding *Streptococcus* abundance [43]. *Streptococcus* isolates are naturally present in the digestive tract [9] but their proportion seems to be higher in CF patients [9]. We identified a significant over-representation of *Streptococcus* spp. in CF children with intestinal inflammation (Figure 3A), with a notable proportion of *S. oralis* (Figure 4). *Streptococcus* strains may contribute to the inflammatory process by synergistic interactions with other commensal microorganisms such as *Candida albicans* or *Veillonella spp.,* both being increased in IBD flares [23,44,45]. Overall, these data support the relationship between respiratory and digestive microbiomes.

This CF “gut–lung axis” is poorly described. It appears to be bidirectional with intestinal microbiota changes predicting pulmonary colonization of *Pseudomonas aeruginosa* [40] and oral probiotics decreasing pulmonary exacerbations [46]. Interestingly, we found that intestinal inflammation at the inclusion was associated with an increase in short-term antibiotic cures, especially with an increase over the next two years in inhaled antibiotic courses (Table 1). However, in the absence of repeated fecal calprotectin measurement during these two years, we cannot affirm that non-inflammatory patients at baseline remain without intestinal inflammation during this period.

Short-term intestinal inflammation may affect the nutritional status of CF patients, as demonstrated by significant correlations between fecal calprotectin level and both weight z-scores and height z-scores of CF patients [47]. As previously recorded [21], we did not find a significant correlation between intestinal inflammation and BMI. BMI evolution is recognized as multifactorial and is now part of the nutritional management, fully integrated into CF disease treatment [21].

Long-term inflammation could impact morbidity and mortality, especially regarding the increased risk of small intestine and colon cancers in CF patients compared with the general population [48]. The pathogenesis of digestive cancers in CF remains unclear. It has been shown that chronic intestinal inflammation is associated with this malignancy risk [49]. Furthermore in IBD, risk factor for colorectal cancer is correlated with the intensity and duration of the inflammation [50]. CF inflammation is present even at an early age, as well demonstrated in our pediatric cohort than in previous studies [51,52], which may be a key factor in developing digestive malignancy later on.

Microbiota composition has also been associated with digestive cancer, due to a decrease in bacteria protecting against cancer and changes in the corresponding metabolite production, such as a decrease in butyrate production, also observed in CF patients with intestinal inflammation [49,53,54]. We and others [10,22,40,54,55] showed that *Bacteroides*—known as protective bacteria against malignancy [53]—are decreased in CF.

## 5. Conclusions

To conclude, with more CF patients surviving into advanced adulthood we need to improve our understanding and management of this systemic disease, including the chronic intestinal inflammation. Few studies including ours reinforce the relation between intestinal inflammation and microbiota disturbance. Further in vitro and in vivo studies are warranted given the limited understanding of the CF physiopathology. Even well-documented probiotic strains, such as *Bifidobacterium adolescentis* identified in this study to be significantly decreased—that inhibits inflammatory responses in intestinal epithelial cells [37]—will require further clinical trials to decipher their potential ability to reduce the short-term intestinal inflammation and the long-term cancer risk. 

## Figures and Tables

**Table 1:** Characteristics of patients with and without intestinal inflammation.

	No Inflammatory Group	Inflammatory Group
**Patient**	13 (65%)	7 (35%)
**Fecal calprotectin level**	122 (91.0–149.0)	459 (324.5–925.0)
**Age in years**	9 (7.0–11.0)	8 (7.5-11.5)
**Female**	7 (53.9%)	3 (43%)
**Mutations**		
- F508del homozygous	10 (77%)	4 (57%)
- F508del heterozygous	2 (15%)	3 (43%)
- Others	1 (8%)	0
**%BMI ^†^**	97.6 (94.0–108.0)	98.3 (88.5–97.7)
**%FEV_1_^††^**	81(71.0–91.0)	76 (71.5–93.5)
**Chronic pulmonary colonization**		
- *P. aeruginosa*	1 (8%)	0
- *S. aureus*	11 (85%)	7 (100%)
Previous IV antibiotic courses *	0 (0–2)	5 (0.5–10)
**Quality of life**		
- Parents’ report	81.9 (76.5–88.4)	89.0 (86.7–90.4)
- Child’s report	83.9 (71.9–89.2)	85.1 (76.8–88.6)
**Digestive symptoms ^••^**		
- Parents’ report	83.2 (80.6–90.7)	94.5 (93.6–95.0)
- Child’s report	84.5 (79.4–95.1)	91.2 (88.7–98.1)
**Follow-up at 2 years**		
- %BMI ^†^	96.6 (90.6–105.9)	97.6 (92.3–102.6)
- %BMI variation	−0.5 (−3.8–1.5)	−0.3 (-1.0–3.0)
- %FEV_1_^††^	84.0 (71.2–95.2)	83.5 (69.5–85.5)
- %FEV_1_ variation	1 (−21.0–8.5)	9 (-4.7–14.5)
- IV antibiotics	2.5 (0.0–5.2)	6.5 (5.2–9.2)
- Oral antibiotics	1.5 (0.0–3.2)	2 (2.0–2.7)
- Inhaled antibiotics +	1 (1.0–1.2)	2 (2.0–2.7)
- Total antibiotics +	5 (3.7–8.2)	11.5 (10.2–12.7)

Data are presented as n (%) or median (interquartile interval); Abbreviations: BMI, body mass index; FEV_1_, Forced Expiratory Volume in 1s; IV, intravenous; + *p* < 0.05; ^†^ expressed as percent of the standard normalized by age; ^††^ expressed as percent predicted; * Evaluated using PedsQL^TM^ Generic Core Scale 4.0; ^••^ Evaluated using PedsQL™ Gastrointestinal Symptoms Scales 3.0.

## Supplementary Materials

The following are available online at https://www.mdpi.com/2077-0383/8/5/645/s1. Table S1: Clinical information for 20 CF patients; Table S2: Significant differentially present taxa between patients with low and high calprotectin levels. Table S3: Results of LEfSe for patients with low and high calprotectin levels. Table S4: Index of dysbiosis (MD-index) per patient. Supplemental information: Additional informations.
