# Supplementary material for: Intestinal Inflammation in Children with Cystic Fibrosis Is Associated with Crohn’s-Like Microbiota Disturbances
Source: J Clin Med. 2019 May 10;8(5):645. doi: 10.3390/jcm8050645 (PMC6572243; doi:10.3390/jcm8050645)
Supplement: Supplementary file 1 [file jcm-08-00645-s001.zip › Supplemental information.docx]

**Supplemental information**

**Methods**

**Absolute quantification of *Streptococcus oralis* and total bacteria using droplet digital PCR (ddPCR)**

The quantitative analysis of *S. oralis* and total bacterial populations was performed on each DNA extracted from fecal samples by ddPCR. Briefly, mixture containing 11 µL ddPCR™ Supermix for Probes (no dUTP) (Bio-Rad, ‎Hercules, California‎, USA), 2.2 µL of 7 µM forward primers (AATCAATACTATCGCCCTGTGCTT), 2.2 µL of 7 µM reverse primers (AGCGATTGCGGCACTTCT), 2.2 µL of 7 µM probes (6-carboxyfluorescein (FAM)-AGATGGCATTTCCCGCTATCTAACTGACG-ZEN/Iowa Black^®^ FQ) (IDT, Coralville, IA, USA) [51], 2.4 µL of Milli-Q water (Millipore Corporation, Burlington, Massachusetts, USA), and 2µL of extracted DNA was used for amplifying gtfR gene of *S. oralis*, ddPCR mixture for total bacterial 16S rRNA quantification was composed of 11 µL ddPCR™ Supermix for Probes (no dUTP), 2.2 µL of 9 µM forward primers (CGGTGAATACGTTCCCGG), 2.2 µL of 9 µM reverse primers (TACGGCTACCTTGTTACGACTT), 2.2 µL of 9 µM probe (6-carboxyfluorescein (FAM)-CTTGTACACACCGCCCGTC-ZEN/Iowa Black^®^ FQ) (IDT, Coralville, IA, USA) [52], 2.4 µL of Milli-Q water and 2µL of DNA. A volume of 20 µL of each reaction mix-sample was loaded into the QX200 droplet generator (Bio-Rad, ‎Hercules, California‎, USA) and droplets were formed according to manufacturer's instructions. The droplets were transferred onto 96-well PCR plates (Eppendorf), and amplified on a C1000 thermal cycler with a 96-deep-well reaction module (Bio-Rad), under the following conditions. Each reaction began with denaturation for 5 min at 95°C, followed by 40 cycles of 95°C for 30 s and 60°C for 1 min, and a final step of 10 min at 98°C. Droplets were analyzed immediately via QX200^TM^ Droplet Reader^TM^ with Quantasoft software v. 1.7.4 ((Bio-Rad, ‎Hercules, California‎, USA). For each DNA sample, proportion of *S. oralis* was estimated using the log of ratio between *S. oralis* and 16S rRNA quantification by ddPCR.
